# Impact of physical activity on anxiety among university students: a moderated mediation model

**DOI:** 10.3389/fpsyg.2024.1509201

**Published:** 2024-11-18

**Authors:** Tianci Qin, Ping Chen, Jiale Wang, Junwei Dong, Kai Zhang

**Affiliations:** School of Sports Science, Jishou University, Jishou, China

**Keywords:** physical activity, mental toughness, social support, anxiety, university students

## Abstract

Physical activity among university students significantly influences anxiety, yet the underlying mechanisms require further exploration. This study examines mental toughness as a mediator and social support as a moderator to elucidate these relationships.

**Methods:**

Using a cross-sectional design, convenient sampling was employed to select 997 first to fourth-year students from Jishou University for a self-reported survey. Measures included assessments of physical activity, mental toughness, social support, anxiety, and basic demographic variables. Descriptive statistics, correlations, and a moderated mediation model were conducted.

**Results:**

Physical activity was significantly negatively correlated with anxiety and positively correlated with mental toughness. Mental toughness was significantly negatively correlated with anxiety, mediating the relationship between physical activity and anxiety to a certain extent. Social support moderated the latter part of the mediated model pathways.

**Conclusion:**

Physical activity negatively correlates with anxiety among university students. Mental toughness mediates the relationship between physical activity and anxiety, while social support moderates the latter stages of this mediated model (mental toughness → anxiety).

## Introduction

Anxiety is an emotional and physiological state characterized by feelings of tension, helplessness, and fear in response to unknown or uncertain events (Knight et al., 2019). Currently, university students face various stresses such as entrance exams, interpersonal relationships, and future careers, making them susceptible to negative emotions like anxiety (Zhan et al., 2021). In China, anxiety among university students is prevalent, with detection rates continuously rising and remaining alarmingly high, reaching up to 25.7% (Cheng and Jia, 2019; Yu and Huang, 2023). Research indicates that anxiety is often a risk factor for engaging in behaviors such as aggression, suicidal ideation, smoking, alcohol consumption, dieting, and internet addiction (Wiebenga et al., 2022; Binod, 2020; Ghahremani et al., 2019; Wang et al., 2022; Kumar et al., 2022). Failure to alleviate anxiety during this period can significantly impact both physical and mental health, increasing the risk of developing mental disorders in adulthood (Zhu, 2021).

Addressing this issue, studies suggest a correlation between physical activity and anxiety. Physical activity (Caspersen et al., 1985) refers to any bodily movement generated by skeletal muscle contraction resulting in energy expenditure. It not only promotes physical health but also exerts positive effects on psychological well-being. Engaging in sustained appropriate physical activity contributes to higher levels of psychological health and inhibits further anxiety development (Schuch and Vancampfort, 2021). Experimental research underscores the significant role of physical activity in reducing anxiety levels among university students (Murray et al., 2022). A meta-analysis (Lin and Gao, 2023) confirms that physical activity significantly improves depression and anxiety, serving as an effective intervention for enhancing psychological health. According to the hypothalamic–pituitary–adrenal (HPA) axis regulation theory (Herman et al., 2020), the HPA axis is a crucial component of the neuroendocrine system that regulates individual emotions and cognition (Raymond et al., 2018). Physical activity can mitigate stress responses by regulating the HPA axis, thereby alleviating anxiety (Mahmood et al., 2020). Based on the above review, this study hypothesized that physical activity significantly negatively predicted the occurrence of anxiety.

Despite this, two significant questions remain largely unanswered. The first concerns the mechanisms through which physical activity may reduce the risk of anxiety among university students. The second question addresses whether protective factors, such as social support, can mitigate the adverse effects of low mental toughness on anxiety in this population. Answers to these questions are crucial, as they will elucidate the complex and implicit relationship between physical activity and anxiety, and provide a foundation for preventive and intervention strategies aimed at reducing anxiety among university students.

Furthermore, engaging in appropriate physical activity enhances mental toughness (Belcher et al., 2021), which may mitigate the occurrence of negative emotions such as anxiety. Previous research indicates that mental toughness (Sun et al., 2019; Li et al., 2020) represents an individual’s ability and trait to adapt well or thrive in the face of adversity. Mental toughness significantly inhibits anxiety, especially in dimensions such as family support, emotional control, and interpersonal assistance (Fang et al., 2022). According to the mental toughness process model (Richardson, 2002), when individuals face stressful or adverse life events beyond their defense systems’ capability, their level of mental toughness decreases, leading to an imbalanced state and negative emotions. As an external protective factor, engaging in physical activity actively contributes to enhancing mental toughness (San Román-Mata et al., 2020), fostering a positive mindset in coping with adversity, reducing stress, restoring balance, and mitigating the occurrence of fear and anxiety (Wan et al., 2024). Based on this evidence, we hypothesize that mental toughness may play a mediating role in the effect of physical activity on anxiety.

However, the strength of the relationships among these variables varies when individuals are influenced by various internal and external factors, with social support being a particularly significant factor (Mecha et al., 2023). Research indicates that social support acts as an external protective factor for mental toughness, encompassing various forms of material assistance, life sustenance, and emotional comfort received within social relationships (House, 1987). It serves as a crucial indicator predicting mental health, as higher levels of social support correspond to increased mental toughness (Yıldırım and Tanrıverdi, 2021). Moreover, social support significantly negatively predicts anxiety; individuals receiving less social support are more prone to anxiety, whereas those with greater social support experience lower anxiety levels (Ren et al., 2020). The buffering effect model of social support (Kerres Malecki and Kilpatrick Demary, 2002) suggests that when individuals face stress, anxiety, or other negative life events, the presence of social support influences these events to some extent. Sufficient social support can mitigate adverse reactions, promote favorable adjustments, and thereby buffer negative emotions, enhancing individual mental health and reducing anxiety. Therefore, this study hypothesizes that social support moderates the latter part of the pathway through which physical activity influences anxiety via mental toughness as a mediator.

**Figure 1:**
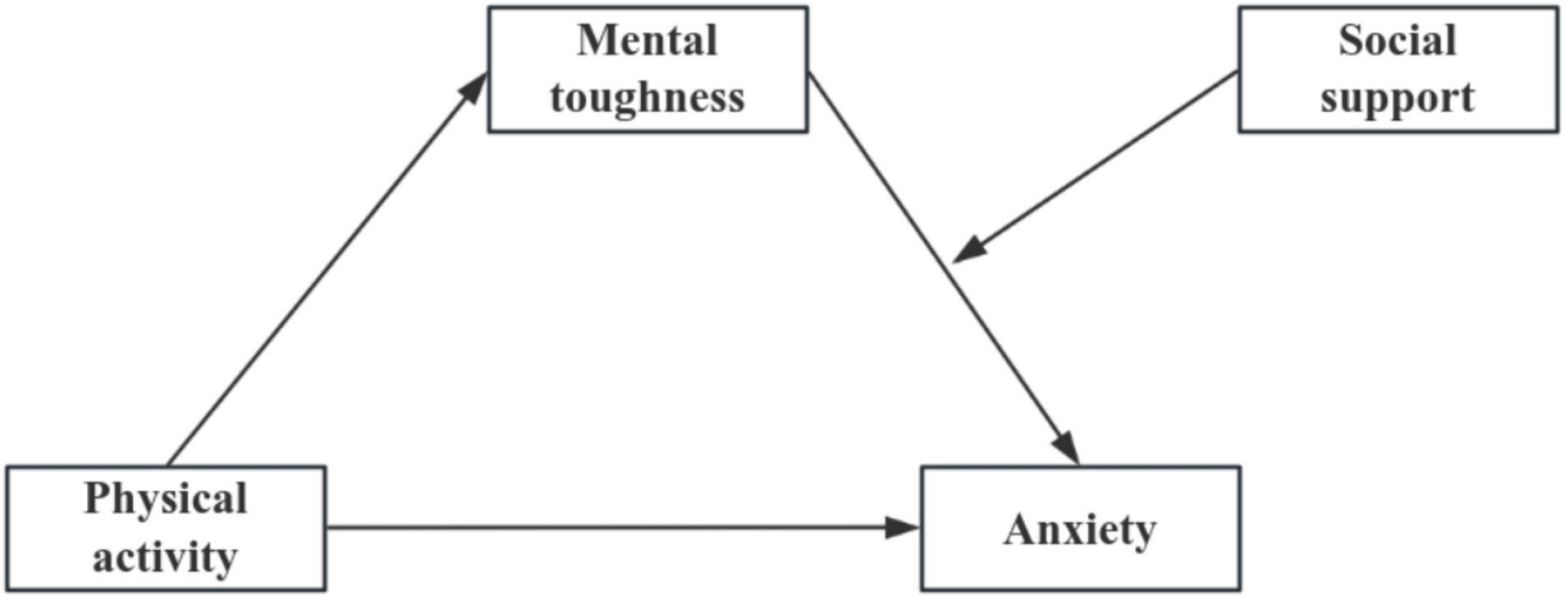
Mediated model assumptions.

In conclusion, this study focuses on Chinese university students, To explore the relationship between physical activity and anxiety, establishing a mediation model to explore the mediating effect of mental toughness between physical activity and anxiety. Furthermore, it investigates the moderating role of social support in the relationships among physical activity, mental toughness, and anxiety. This study aims to construct a moderated mediation model (see Figure 1).

## Materials and methods

### Participants

Based on the study by Jin (2023), where the prevalence (P) of anxiety among college students was found to be 29% with a tolerable error (e) of 3% and a confidence level (Z) of 1.96, the required sample size calculation indicates a minimum of 880 surveys.

The sample size calculation formula is:

$$n=Z^{2}\frac{P\left(1-P\right)}{e^{2}}$$

where (n) represents the sample size, (Z) is the confidence level, (e) is the tolerable error, and (P) is the probability.

This cross-sectional survey was conducted in June 2024. Following the principle of convenient sampling, a questionnaire survey was conducted among college students in Jishou City, Hunan Province. In this study, online electronic questionnaires were distributed through class groups, unified training was conducted for the distribution personnel before the questionnaire was distributed, and questionnaires were distributed through teachers of each class. The subjects were given questionnaires in the class group by the teacher. The teacher first introduced to the subjects the main purpose of the study, the anonymity of the data and the final destination of the data, and the survey had no risk to the research subjects. All participants had obtained online informed consent, and then the electronic questionnaires were issued. Participants completed the electronic questionnaire in about 15 min, and we set a reasonable minimum response time based on the length and complexity of the questionnaire. The responses were considered too short to ensure that participants could complete the questionnaire carefully. A total of 1,081 questionnaires were sent out, and 997 were recovered after excluding invalid questionnaires, with an effective recovery rate of 92.23%. The final analysis sample of this study included 997 college students (562 males and 435 females; 360 freshmen, 257 sophomores; 192 students in 3 years; Senior 188 students; Liberal arts 262 students, science 611 students, other 124 students); Only child (family with only one child): 274, non-only child (family with two or more children): 723.

## Methods

### Basic information questionnaire

A demographic survey was used to collect population variables including gender, academic year, only-child status, and long-term residence (urban or rural). These demographic variables were controlled for in the analysis due to their potential influence on study outcomes.

### International physical activity questionnaire (IPAQ)

The International Physical Activity Questionnaire (IPAQ) (Qu and Li, 2004), was employed to assess physical activity across six dimensions (work-related activities, commuting, household chores, leisure time physical activity and exercise, sedentary time, and sleep). Data cleaning and truncation procedures were applied: each physical activity’s daily duration was converted to minutes, activities missing duration or frequency data were excluded from analysis. Activities exceeding 180 min per day were truncated to 180 min, and weekly totals exceeding 1,260 min were truncated to 1,260 min. Participants reporting total daily physical activity exceeding 960 min were excluded from analysis. According to Table A1, the calculated physical activity amount of individuals was divided into three types of physical activity levels: high, medium and low (see Tables A1, A2 in annex).

### Connor-Davidson mental toughness scale (CD-RISC)

mental toughness among students was measured using the Chinese version of the Connor-Davidson Mental toughness Scale (CD-RISC) (Yu and Zhang, 2007). This scale consists of 25 items assessing mental toughness across dimensions of toughness, strength, and optimism. Scores range from 0 (not true at all) to 4 (true nearly all the time), with higher total scores indicating greater mental toughness. Internal consistency in this study was excellent (Cronbach’s *α* = 0.970).

### Perceived social support scale (PSSS)

The Perceived Social Support Scale (PSSS), developed by Zimet et al. (1990) and revised by Huang et al. (1996), comprises 12 items measuring subjective perceptions of support from family, friends, and others. Responses range from 1 (very strongly disagree) to 7 (very strongly agree) using Likert scaling. An example item includes: “I can rely on my friends when things go wrong.” Higher scores indicate greater perceived social support. Internal consistency in this study was high (Cronbach’s *α* = 0.952).

### Generalized anxiety disorder scale (GAD-7)

Anxiety symptoms were assessed using the Generalized Anxiety Disorder Scale (GAD-7) (Spitzer et al., 2006). This 7-item scale measures anxiety severity on a scale from 0 (not at all) to 4 (nearly every day). Total scores categorize anxiety levels as: 0–4 (none), 5–9 (mild), 10–14 (moderate), and 15–21 (severe). Internal consistency in this study was high (Cronbach’s *α* = 0.945).

### Statistical analysis

Data collected from the questionnaires were entered into Excel and analyzed using SPSS 26.0. Normality was assessed using the Kolmogorov–Smirnov test (K-S test) and Harman’s single-factor test for common method bias. Reliability and validity of study tools were evaluated through reliability analysis and factor analysis. Descriptive statistics were used to summarize demographic variables and study variables. Spearman correlation analysis examined associations between physical activity, mental toughness, social support, and anxiety. The PROCESS 4.0 plugin for SPSS was employed to analyze direct effects of physical activity on anxiety, simple mediation effects of mental toughness, and moderation effects of social support. Indirect effects were tested using Model 4 and Model 14, with 5,000 bootstrap samples to estimate bias-corrected percentile confidence intervals (95% CI). Finally, Origin2022 was used to graphically depict the moderating role of social support on the relationship between mental toughness and anxiety.

#### Assessment of common method bias

To assess common method bias, the Harman’s single-factor test in SPSS indicated that 13 factors had eigenvalues greater than 1. The first factor explained 28.699% of the variance, below the recommended threshold of 40% (Tang and Wen, 2020). Thus, the study did not exhibit significant common method bias. Normality testing of all variables showed significance levels below 0.05, indicating non-normal distribution of the data. Consequently, non-parametric tests were employed for all subsequent analyses.

## Results

### Descriptive statistical analysis of survey sample variables

To evaluate physical activity, different activity types were converted into weekly expenditure of MET-minutes. Descriptive statistics were utilized to summarize the primary variables and their sub-dimensions. As illustrated in Table 1, the median total physical activity expenditure for college students was 2,374 MET-minutes per week. Specifically, the median expenditure for recreational physical activities was 1,095 MET-minutes per week, for transportation-related physical activities was 656 MET-minutes per week, for daily living activities was 180 MET-minutes per week, and for work-related physical activities was 0 MET-minutes per week.

**Table 1:** Describes the statistical analysis of each variable.

Variable	*N* = 997		
	25%	50%	75%
High intensity physical activity	0	540	1,440
Moderate intensity physical activity	80	480	960
Low intensity physical activity	468.8	1,014	1743.5
Daily physical activity at work	0	0	0
Daily traffic physical activity	284.5	656	1321.5
Physical activity in daily life	0	180	570
Sports leisure physical activity	396	1,095	2,236
Total physical activity	1,169	2,374	4,220
Tenacity	25	32	37
self-improvement	14	19	23
Optimistic	7	9	12
Mental toughness	48	61	71
Family support	16	20	24
Peer support	16	20	23
Other support	16	20	24
Social support	49	60	69
Anxiety	2	7	12

These results indicate that 75% of the students did not engage in regular part-time work. Furthermore, 25% of the students did not achieve the recommended level of physical activity for daily living, and 75% did not meet the threshold for high-intensity physical activity. Additionally, the median score for overall mental toughness was 61, the median score for overall social support was 60, and the median score for anxiety was 7, as shown in Table 1.

### Correlation analysis of variables

Table 2 illustrates significant correlations among variables in the study. Specifically: There is a significant positive correlation between physical activity and mental toughness (*r* = 0.278, *p* < 0.01), as well as between physical activity and social support (*r* = 0.356, *p* < 0.01). A significant negative correlation exists between physical activity and anxiety (*r* = −0.299, *p* < 0.01).mental toughness shows a significant positive correlation with social support (*r* = 0.496, *p* < 0.01) and a significant negative correlation with anxiety (*r* = −0.365, *p* < 0.01).

**Table 2:** Correlation test results of all variables.

	Physical activity	Mental toughness	Social support	Anxiety
Physical activity	1.000			
Mental toughness	0.278^**^	1.000		
Social support	0.356^**^	0.496^**^	1.000	
Anxiety	−0.299^**^	−0.365^**^	−0.353^**^	1.000

** means *p* < 0.01, * means *p* < 0.05.

Social support demonstrates a significant negative correlation with anxiety (*r* = −0.353, *p* < 0.01).

### Mediation analysis

Table 3 demonstrates that, controlling for gender and demographic variables, physical activity significantly negatively predicts anxiety among university students (*β* = −0.298, *t* = −9.806, *p* < 0.001). When mental toughness serves as a mediator, physical activity continues to significantly negatively predict anxiety (*β* = −0.237, *t* = −8.260, *p* < 0.001). Furthermore, in Model 2, physical activity significantly positively predicts mental toughness (*β* = 0.171, *t* = 5.464, *p* < 0.001), and the mediating variable mental toughness significantly negatively predicts anxiety (*β* = −0.355, *t* = −12.389, *p* < 0.001).

**Table 3:** Test of mediation effect model.

	Model 1 (Anxiety)			Model 2 (Mental toughness)			Model 3 (Anxiety)		
	β	SE	t	β	SE	t	β	SE	t
Sex	−0.007	0.064	−0.214	0.032	0.066	0.487	−0.001	0.060	−0.038
Physical activity	−0.298	0.030	−9.806***	0.171	0.031	5.464***	−0.237	0.029	−8.260***
Mental toughness							−0.355	0.029	−12.389***
R^2^	0.089			0.030			0.212		
F	16.135***			5.167***			37.887***		

^*^P < 0.05, ^**^P < 0.01, ^***^P < 0.001.

To further validate these findings, the mediation of mental toughness between physical activity and anxiety was examined using standardized coefficient Bootstrap tests. The data were first standardized, followed by testing the mediation effect, as presented in Table 4.

**Table 4:** Total effect, direct effect and intermediate effect breakdown table.

	Effect size	BootSE	BootCI95% confidence interval		Percentage
			Lower limit	Upper limit	
Total effect	−0.298	0.026	−0.351	−0.248	
Direct effect	−0.237	0.024	−0.283	−0.188	79.59%
Indirect effect	−0.061	0.018	−0.101	−0.030	20.41%

**Figure 2:**
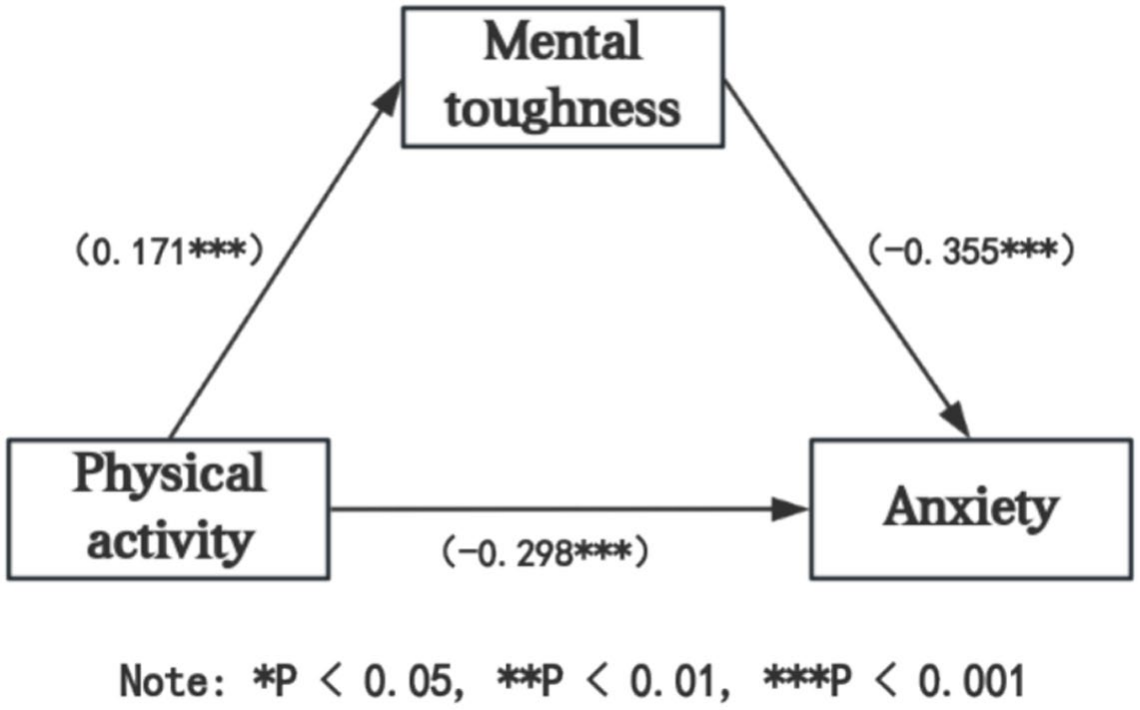
Mediation model diagram.

From Table 4, it is evident that the 95% Bootstrap confidence intervals for both the direct effect of physical activity on anxiety and the indirect effect through mental toughness (see Table 5) do not include zero. This indicates that physical activity not only directly predicts anxiety but also indirectly predicts anxiety through the mediating effect of mental toughness. The direct effect (−0.237) has a confidence interval of [−0.283, −0.188], while the mediation effect of mental toughness (−0.061) has a confidence interval of [−0.101, −0.030]. These effects, respectively, constitute 79.59 and 20.41% of the total effect (−0.298), with a confidence interval of [−0.351, −0.248]. Detailed pathways of the effects are illustrated in Figure 2.

**Table 5:** Overall moderated mediating effect.

Index	BootSE	BootCI95% confidence interval	
		Lower limit	Upper limit
0.015	0.006	0.006	0.028

### Moderated mediation model testing

We employed Model 14 from the Process 4.0 plugin developed by Hayes (2009) to examine moderated mediation effects, controlling for covariates. To mitigate non-essential multicollinearity and enhance interpretability of results, primary variables were standardized. We calculated the interaction term between the standardized mediator and moderator variables. Detailed examination results are presented in Table 6.

**Table 6:** Moderated mediation model tests.

Variable	Model 1 Mental toughness			Model 2 Anxiety		
	*β*	SE	*t*	*β*	SE	*t*
Sex	0.032	0.066	0.487	−0.003	0.058	−0.049
Physical activity	0.171	0.031	5.464***	−0.168	0.029	−5.711***
Mental toughness				−0.238	0.032	−7.363***
Social support				−0.172	0.034	−5.030***
Mental toughness*Social support				0.089	0.020	4.404***
R	0.174			0.505		
R2	0.030			0.255		
F	5.167***			37.466***		

*P < 0.05, **P < 0.01, ***P < 0.001.

**Figure 3:**
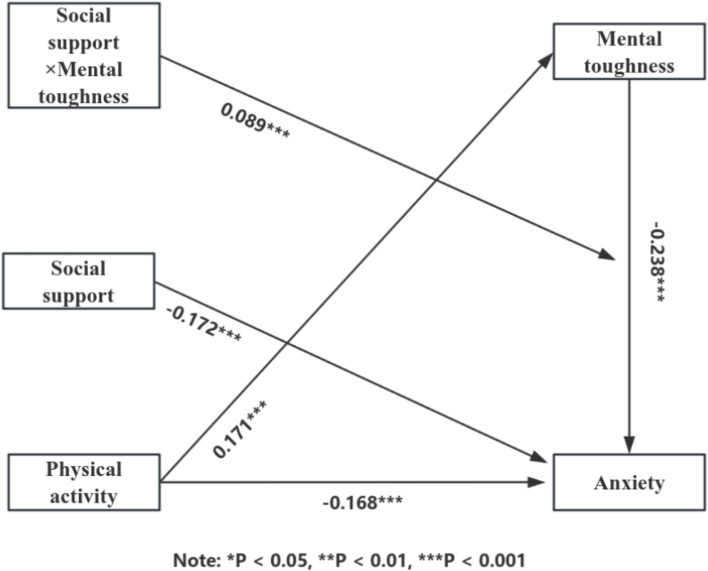
Moderated mediating effect model diagram.

According to the results presented in Table 6, in Model 1, physical activity had a significant effect on mental toughness (*β* = 0.171, *t* = 5.464, *p* < 0.001). In Model 2, physical activity (*β* = −0.168, *t* = −5.711, *p* < 0.001), mental toughness (*β* = −0.238, *t* = −7.363, *p* < 0.001), and social support (*β* = −0.172, *t* = −5.030, *p* < 0.01) all significantly influenced anxiety. The interaction between mental toughness and social support (*β* = 0.089, *t* = 4.404, *p* < 0.001) also significantly predicted anxiety. This suggests that social support moderates the impact of mental toughness on anxiety. The model diagram is depicted in Figure 3.

Table 7 indicates that at low levels of social support, the mediation effect of mental toughness on anxiety is significant, with a direct effect of −0.056 (Bootstrap SE = 0.017) and a confidence interval of [−0.093, −0.027], which does not include zero. Similarly, at high levels of social support, the mediation effect of mental toughness is also significant, with an effect of −0.026 (Bootstrap SE = 0.012) and a confidence interval of [−0.053, −0.006], which does not include zero. The mediation effect is greater at low levels of social support compared to high levels.

**Table 7:** The mediating role of mental toughness between physical activity and anxiety at different levels of social support.

Social support	Intermediate effect size	BootSE	BootCI95% confidence interval	
			Lower limit	Upper limit
M-1SD	−0.056	0.017	−0.093	−0.027
M	−0.041	0.013	−0.071	−0.018
M + 1SD	−0.026	0.012	−0.053	−0.006

Furthermore, the overall moderated mediation model, as indicated by the confidence interval of the index for Table 5 [0.006, 0.029], which does not include zero, confirms the significant overall effect and supports the proposed moderated mediation model in this study.

To further clarify the moderating effect of different levels of social support on the relationship between mental toughness and anxiety, a simple slope analysis was conducted. Social support and mental toughness were grouped based on one standard deviation above and below the mean, as shown in Table 8.

**Table 8:** Effects of mental toughness on anxiety at different levels of social.

Social support	Effect size	SE	*t*	*p*	LLCI	ULCI
M-1SD	−0.327	0.035	−9.269	0.000	−0.396	−0.258
M	−0.238	0.032	−7.363	0.000	−0.301	−0.174
M + 1SD	−0.149	0.041	−3.654	0.000	−0.229	−0.069

**Figure 4:**
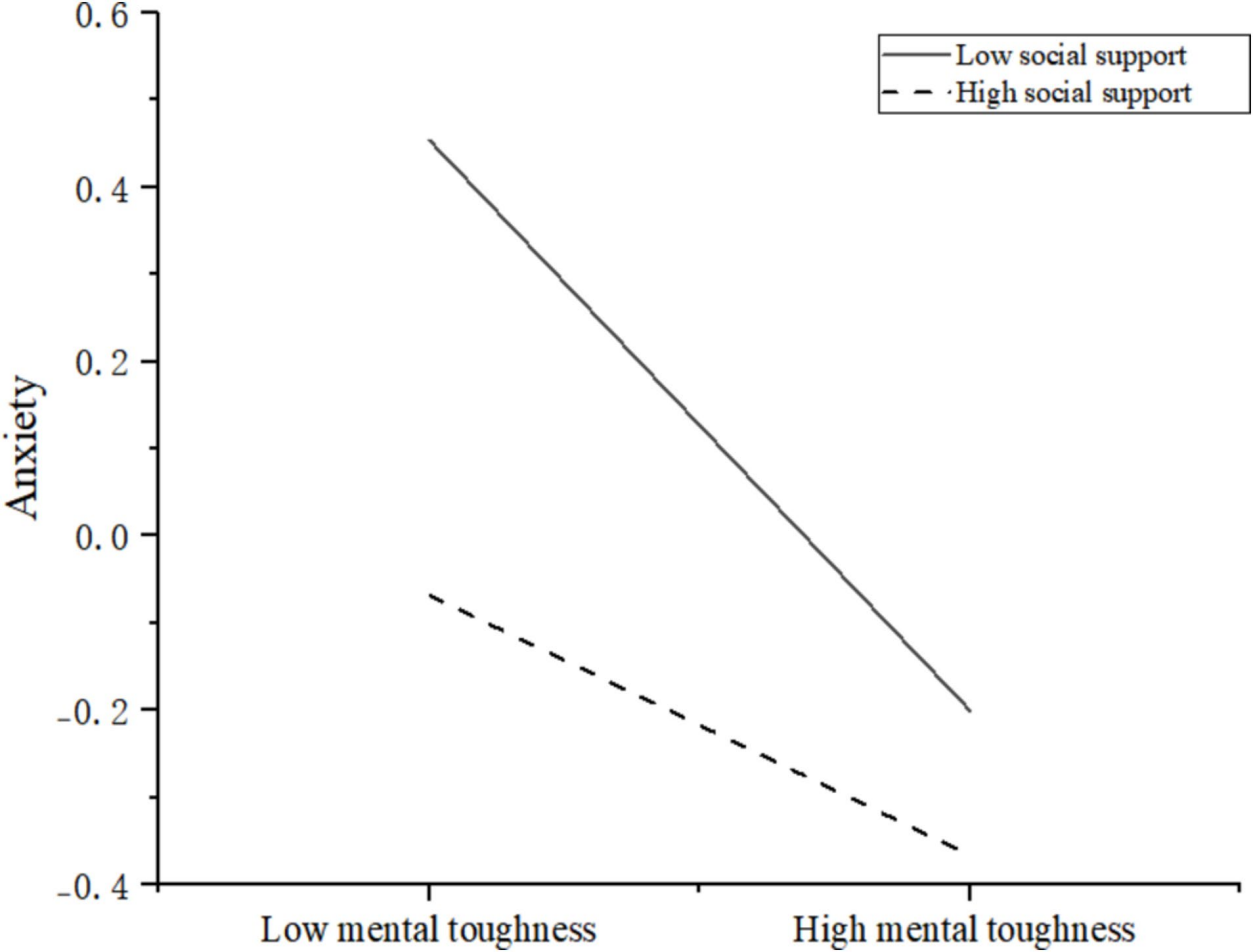
A simple slope analysis of the interaction between social support and mental toughness on anxiety.

Table 8 illustrates that at low levels of social support (M-1SD), mental toughness significantly negatively predicts anxiety (effect size = −0.327, *t* = −9.269, *p* < 0.001). Conversely, at high levels of social support (M + 1SD), mental toughness also significantly negatively predicts anxiety (effect size = −0.149, *t* = −3.654, *p* < 0.001). We utilized Origin 2024 to generate a simple slope analysis plot, detailed in Figure 4.

## Discussion

This study examined the relationships among physical activity, mental toughness, social support, and anxiety among college students. The results indicate a positive correlation between physical activity and mental toughness, and a negative correlation between physical activity and anxiety. Mental toughness also shows a negative correlation with anxiety, all of which are statistically significant. Controlling for demographic variables, mental toughness was found to mediate the relationship between physical activity and anxiety, while social support attenuated the relationship between mental toughness and anxiety, confirming our initial hypotheses.

### Impact of physical activity on anxiety

The findings of this study reveal a significant negative correlation between physical activity and anxiety, consistent with previous research (Kandola and Stubbs, 2020). Based on the endorphin hypothesis (Chen and Nakagawa, 2023), endorphins are amino compounds released by the pituitary gland and hypothalamus that act similarly to morphine or opioids, reducing pain, enhancing mood, and inducing feelings of pleasure and exhilaration. Engaging in regular appropriate physical activity facilitates endorphin secretion (Thorén et al., 1990), reduces muscle pain (Ghosh et al., 2024), enhances endurance, regulates individual emotions (Ali et al., 2021), increases excitability (Pahlavani, 2023), thereby aiding in reducing negative emotions such as anxiety and depression (Alizadeh, 2024), and promoting overall mental and physical health. A cross-sectional study (Liu, 2020) demonstrated that compared to lower levels of physical activity, higher levels of sports engagement among college students were associated with lower scores of anxiety and depression, thereby exerting a positive influence on mental and physical health development. Researchers abroad (Liśkiewicz et al., 2020) conducted metabolomic analyses on the hippocampus and prefrontal cortex, concluding that physical activity promotes the synthesis of endogenous fatty acids in the brain, and increases the expression of hippocampal fatty acids, which alleviate anxiety levels. Furthermore, previous studies (He, 2022) conducted a 12-week intervention involving 1 h of simplified Tai Chi exercises per week with 60 special police officers, finding that increased levels of physical activity among special police officers led to earlier sleep times, thereby alleviating symptoms of anxiety. Further research indicated that 6 weeks of Tai Chi exercises significantly improved anxiety levels among special police officers.

### Mediating role of mental toughness in the impact of physical activity on anxiety

Previous research has consistently shown a positive correlation between physical activity and mental toughness, as well as a negative correlation between mental toughness and anxiety (To et al., 2022; Petzold et al., 2020; Kang et al., 2018). Our study corroborates these findings. Additionally, our experiments support the hypothesis that mental toughness partially mediates the impact of physical activity on anxiety, aligning with previous research (Serrão et al., 2021). Research has shown (Sun, 2018) demonstrated that adolescents with high mental toughness tend to exhibit stronger abilities to cope with setbacks and negative emotions when faced with adverse life events, thereby reducing the occurrence of anxiety and facilitating quicker recovery to a normal psychological state. Furthermore, engaging in physical activity contributes to the enhancement of mental toughness. In competitive sports, good mental toughness serves as a foundation for achieving better performance outcomes, thus highlighting the mutually reinforcing relationship between physical activity and mental toughness (Guszkowska and Wójcik, 2021). According to the mental toughness Compensation Model (Garmezy et al., 1984), physical activity acts as a protective factor for mental health not directly combating anxiety, but indirectly through the enhancement of mental toughness, thereby mitigating the onset of anxiety. College students with lower levels of mental toughness are more susceptible to anxiety-related issues (Wu et al., 2020).

### The moderating role of social support in the relationship between mental toughness and anxiety

This study demonstrates the moderating role of social support in the latter part of the pathway, showing that social support can influence the strength of the relationship between mental toughness and anxiety. Mental toughness is identified as an intrinsic protective factor within individuals, while social support serves as an external protective factor. Both factors positively impact an individual’s psychological well-being (Simon et al., 2021). The correlational analysis in this study indicates that higher levels of mental toughness correlate with higher levels of social support. According to the theoretical framework of mental toughness (Kumpfer, 2002), social support is one of the external environmental protective factors contributing to mental toughness. Enhanced social support facilitates higher levels of mental toughness, promoting stable emotional states and reducing anxiety. Research by domestic scholars (Sun, 2021) suggests that middle school students benefit from external care and support from peers, parents, and teachers, thereby enhancing their psychological mechanisms, increasing mental toughness levels, and mitigating anxiety during stressful situations such as exams. A cross-lag analysis indicates that providing appropriate social support and interventions to maintenance hemodialysis patients can enhance their mental toughness levels and foster mental health development (Wang et al., 2024). Additionally, studies (Zhou, 2022) suggest that high school students facing external pressures can bolster their confidence in handling difficulties through increased social support, which elevates mental toughness levels and reduces anxiety. The main effects model of social support (Cohen and Wills, 1985) proposes that during adversity, adequate social support helps individuals cope effectively, providing material or emotional comfort, enhancing resilience, reducing anxiety, and improving overall well-being. Furthermore, a cross-sectional study abroad (Jamil and Su, 2024) indicates that multidimensional social support from family, friends, and partners alleviates the risks of anxiety and depression. Thus, the above studies confirm our hypothesis that social support can mitigate the predictive impact of mental toughness on anxiety.

### Implications for research and practice

Firstly, this study provides valuable insights for the development of psychological courses in higher education institutions. It aims to enhance students’ physical fitness, promote mental well-being, and mitigate the impact of negative emotions. Additionally, by examining the role of mental toughness as a mediating variable and social support as a moderating variable in the relationship between physical activity and anxiety among university students, this research seeks to understand the specific mechanisms through which mental toughness and social support operate. It is intended to foster a resilient personality in students, enabling them to face difficulties with a positive mindset. Particularly, it encourages students to proactively manage their emotions and improve self-regulation skills. This, in turn, will contribute to a better understanding of the factors influencing and mechanisms behind anxiety in students, thus enhancing the prevention and intervention strategies for anxiety among university students.

### Limitations and suggestions for further research

There are still some shortcomings in this study. Firstly, this study utilized a cross-sectional research design, which may introduce biases in the relationships between variables, and the measured variables are subject to dynamic changes over time, suggesting the need for longitudinal studies in the future. Secondly, the study participants primarily came from a single institution, potentially limiting its generalizability. Lastly, while this study integrated mental toughness and social support to explore the impact of physical activity on anxiety among university students, other influencing factors in this pathway may warrant further investigation.

## Conclusion

This study has elucidated the relationships among physical activity, mental toughness, social support, and anxiety. It was confirmed that physical activity was negatively correlated with anxiety, confirming the mediating role of mental toughness and the moderating role of social support in the relationship between mental toughness and anxiety. Based on these findings, it is recommended that families and schools create diversified physical activities for university students to strengthen the connection between social support and mental toughness. This approach can mitigate anxiety and promote the mental health of university students.

## Data Availability

The raw data supporting the conclusions of this article will be made available by the authors, without undue reservation.

## Appendix

**Table A1:** Physical activity attributes and MET values in IPAQ long volume.

Type of physical activity	Physical activity program	Intensity of physical activity	MET assignment
Daily work related	On foot	On foot	3.3
	Medium strength	intermediate	4.0
	High strength	high	8.0
Daily traffic related	By bus	On foot	1.0
	On foot	On foot	3.3
	Ride a bike	intermediate	6.0
Housework related	Medium strength	Intermediate	4.0
	High strength	intermediate	5.5
Exercise and leisure related	On foot	On foot	3.3
	Medium strength	intermediate	4.0
	High strength	high	8.0

**Table A2:** Grouping criteria for physical activity levels.

Grouping	Criterion
High	Meet any one of the following 2 criteria: 1. The weekly total of all kinds of high-intensity physical activities is ≥3d, and the cumulative energy consumption is ≥1500MET-min/w 2. The total amount of physical activity of the three intensity was ≥7d per week, and the total amount of activity was ≥3000MET-min/w
Middle	Meet any one of the following 3 standard parts: 1. Meet all kinds of high-intensity physical activities for at least 20min per day, with a total of ≥3d 2. Meet all kinds of moderate intensity and/or walking activities for at least 30 minutes per day, for a total of ≥5 days 3. The total amount of physical activity of 3 kinds of intensity was ≥5d, and the total weekly activity amount was ≥600MET-min/w
Low	Meet any one of the following 2 standard parts: 1. No activities were reported 2. Some activities were reported, but did not yet meet the medium and high grouping criteria described above
